# Pleural Metastases From Acral Lentiginous Melanoma Diagnosed by Medical Thoracoscopy

**DOI:** 10.1002/rcr2.70531

**Published:** 2026-03-02

**Authors:** Ad Rian Chong, Khai Lip Ng, Nai‐Chien Huan, Nur Husna Mohd Aminudin, Fazilah Hassan, Kasuma Mohamed Nordin

**Affiliations:** ^1^ Division of Respiratory Medicine, Department of Internal Medicine Melaka Hospital Melaka Malaysia; ^2^ Department of Respiratory Medicine Queen Elizabeth Hospital Kota Kinabalu Malaysia; ^3^ Centre for Innovative Pleural Research, Sir Charles Gairdner Hospital Perth Australia; ^4^ School of Medical and Health Sciences Edith Cowan University Perth Australia; ^5^ Department of Pathology Melaka Hospital Melaka Malaysia

**Keywords:** acral lentiginous melanoma, malignant pleural effusion, medical thoracoscopy, pleural metastasis

## Abstract

Acral lentiginous melanoma (ALM) with pleural metastases is rare and under‐recognised. We report an 86‐year‐old woman with progressive dyspnoea and pleuritic chest pain. 18 months ago, she was diagnosed with stage IIC ALM of the right heel, and was treated with wide local excision, but she declined adjuvant therapy. Imaging demonstrated a large left pleural effusion with diffuse pleural thickening, pulmonary mass, and hepatic metastases. Brownish pleural fluid, along with thoracoscopic identification of multiple pigmented nodules, led to a histologically confirmed diagnosis. Talc pleurodesis was performed for symptom control, and best supportive care was pursued. This case highlights the diagnostic value of medical thoracoscopy in identifying pleural melanoma metastases and emphasises the need to consider melanoma recurrence when pigmented effusions are encountered.

## Graphical abstract

We present a rare case of metastatic acral lentiginous melanoma involving the pleural space, in which definitive diagnosis was established by medical thoracoscopy, highlighting its value in evaluating atypical pleural metastases.

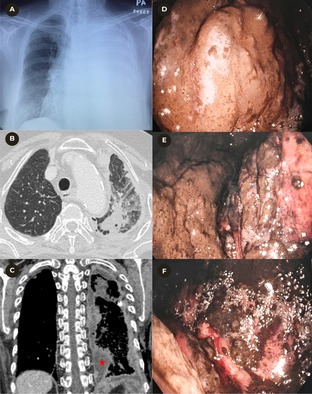

## 1 Introduction

Malignant melanoma is an aggressive neoplasm with a high propensity for distant metastasis, most commonly involving the lungs, liver, brain, and bone [1]. While pulmonary metastases are well recognised, pleural involvement is rare and often underdiagnosed due to nonspecific radiological manifestations such as pleural effusion, thickening, or nodularity, which may mimic other thoracic malignancies including mesothelioma or metastatic lung carcinoma [2, 3].

ALM typically arising on the palms, soles, and nail beds, represents a distinct melanoma subtype associated with delayed diagnosis and a higher risk of advanced visceral dissemination [4]. Reports of pleural metastases arising from ALM are scarce, and detailed descriptions of thoracoscopic findings are limited.

We present a rare case of metastatic ALM involving the pleural space, in which definitive diagnosis was established by medical thoracoscopy, highlighting its value in evaluating atypical pleural metastases.

## 2 Case Report

An 86‐year‐old woman was diagnosed with ALM of the right heel. She underwent wide local excision of the primary lesion with reconstruction using a reverse sural flap and split‐thickness skin graft to achieve adequate wound closure and soft tissue coverage. Postoperative histopathology confirmed ALM with a Breslow thickness of 4.2 mm and the presence of ulceration. Staging investigations, including a computed tomography (CT) scan of the thorax, abdomen, and pelvis, showed no evidence of distant metastasis, consistent with stage IIC melanoma (pT4bN0M0). She declined adjuvant systemic therapy due to concerns regarding toxicity in the context of advanced age.

**FIGURE 1:**
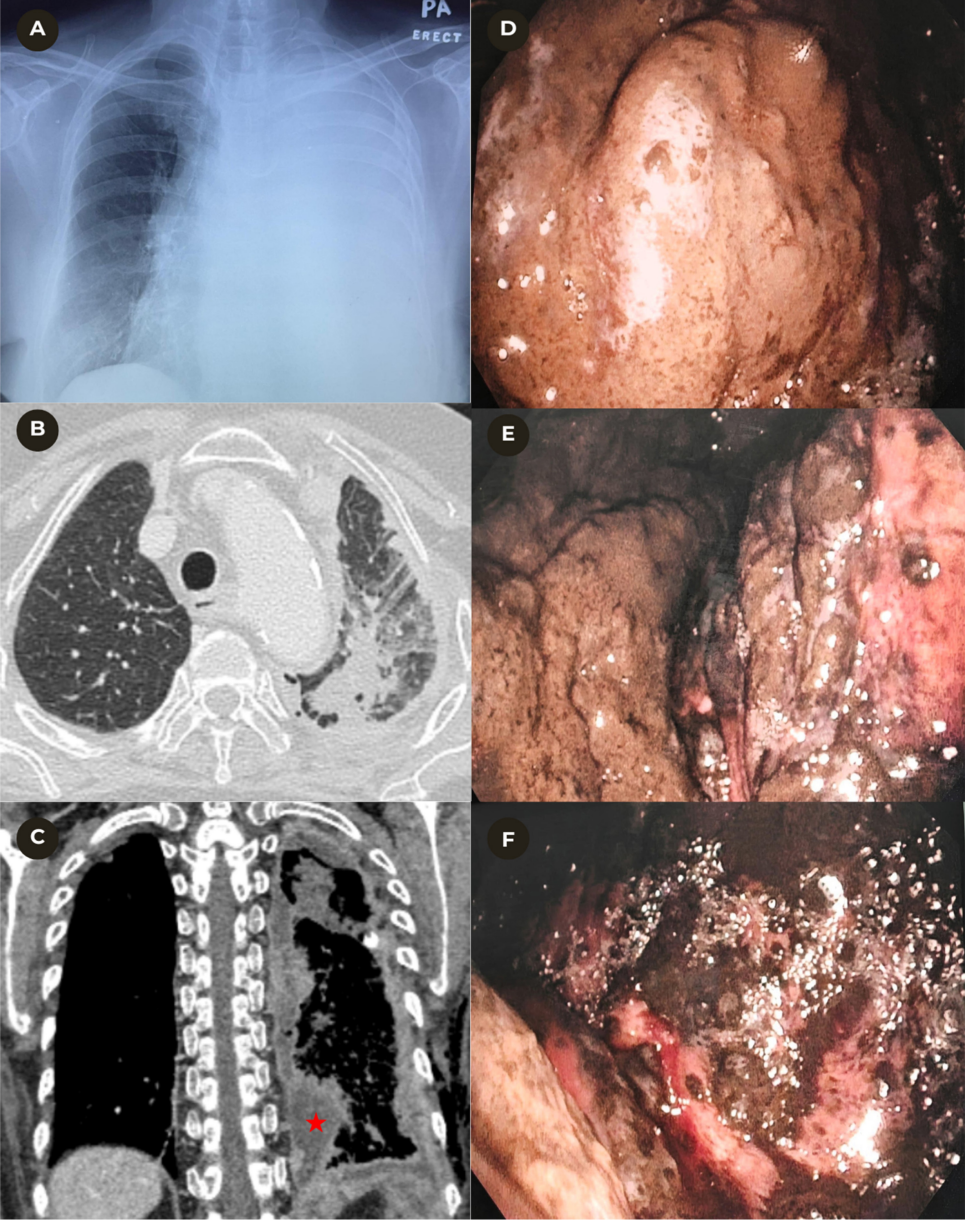
(A) Chest radiograph demonstrating a massive left‐sided pleural effusion. (B) Axial CT thorax (lung window) showing a spiculated 3.3 × 2.4 × 2.6 cm mass in the left apicoposterior segment with interlobular septal thickening and adjacent ground‐glass opacities. (C) Coronal CT thorax (mediastinal window) demonstrating lobulated, enhancing pleural nodules involving the mediastinal and parietal pleura with associated loculated pleural effusion (red star). (D) Thoracoscopic view of the anterior parietal pleura showing nodular pigmented thickening. (E) Thoracoscopic view of the visceral pleura demonstrating nodular pigmented lesions. (F) Thoracoscopic view of the diaphragmatic pleura showing multiple nodular pigmented lesions.

A total of 18 months later, the patient presented to the emergency department with a two‐week history of progressive dyspnoea and left‐sided pleuritic chest pain. Physical examination revealed diminished breath sounds and dullness to percussion over the left hemithorax, and chest radiography confirmed a massive left‐sided pleural effusion (Figure 1A). Thoracentesis yielded brownish pleural fluid that was exudative by Light's criteria; however, cytological examination was non‐diagnostic.

Medical thoracoscopy revealed multiple pigmented nodules studding the parietal and visceral pleura, along with areas of dense pleural thickening (Figure 1D–F). Talc pleurodesis was subsequently performed to prevent recurrence of the effusion and alleviate dyspnoea. Staging CT demonstrated extensive pleural thickening with multiple enhancing nodular lesions throughout the left hemithorax, consistent with metastatic infiltration (Figure 1C). A spiculated 3.3 × 2.4 × 2.6 cm mass was noted in the apicoposterior segment of the left upper lobe, with diffuse interlobular septal thickening and adjacent ground‐glass opacities suggestive of lymphangitic spread (Figure 1B). In addition, multiple hypodense lesions were identified in the liver, consistent with hepatic metastases.

**FIGURE 2:**
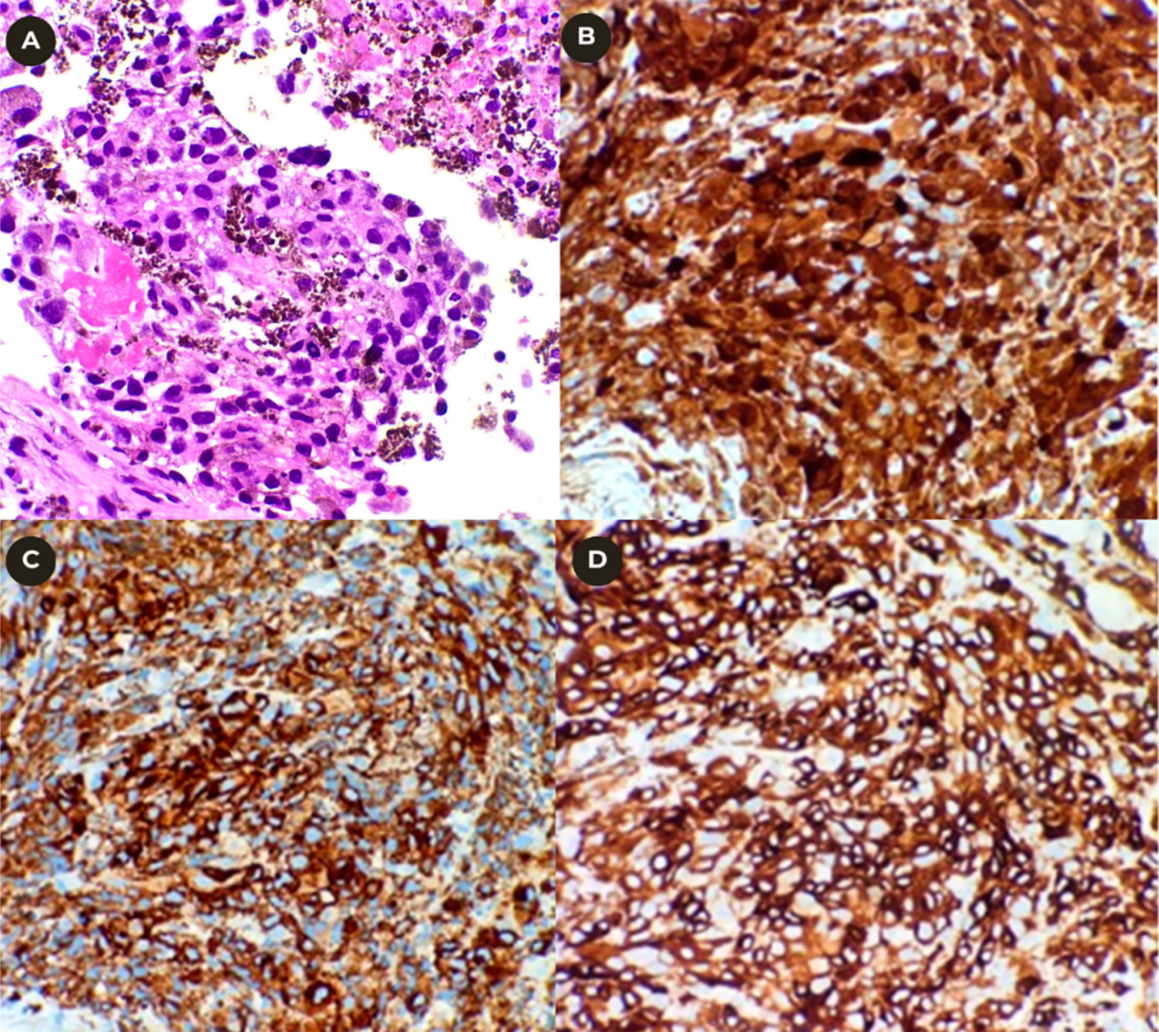
(A) Malignant epithelial cells arranged in sheets with some have abundant intracytoplasmic melanin pigment (haematoxylin and eosin stain, ×400). (B) Immunohistochemical staining positive for S‐100. (C) Immunohistochemical staining positive for Melan‐A. (D) Immunohistochemical staining positive for HMB‐45.

Histopathological examination of pleural biopsies confirmed metastatic melanoma, demonstrating nests of atypical melanocytes with positive immunohistochemical staining for S‐100, Melan‐A, and HMB‐45 (Figure 2A–D). Given the patient's advanced age and the extent of disease, a joint decision was made to pursue best supportive care rather than systemic chemotherapy or immunotherapy.

## 3 Discussion

Malignant melanoma is known for its aggressive biological behaviour and high metastatic potential, yet pleural involvement remains an uncommon site of dissemination. This case is notable for the delayed presentation of extensive pleural metastasis 18 months after definitive surgical management of stage IIC acral lentiginous melanoma, highlighting both the aggressive nature of high‐risk melanoma and the need for continued vigilance during follow‐up [2].

Thoracic metastatic melanoma most commonly manifests with pulmonary involvement, with radiographic studies showing pulmonary metastases in up to 70% of patients with chest dissemination, whereas pleural effusions are reported in only around 2% of cases [5]. In our patient, imaging revealed diffuse pleural thickening, multiple nodular lesions, and a spiculated pulmonary mass with features suggestive of lymphangitic spread, illustrating that melanoma recurrence can present with both parenchymal and pleural involvement, even after an extended disease‐free interval. Melanoma may reach the pleural space through several metastatic routes, including hematogenous dissemination via the bloodstream, lymphatic spread through mediastinal or pleural lymphatics, or direct extension from adjacent pulmonary metastases, contributing to the diverse thoracic presentations observed in advanced disease [6].

Notably, pleural metastatic melanoma may occur after prolonged latency, with cases reported up to 12 years after initial treatment. This delayed presentation is thought to reflect tumour dormancy, whereby disseminated melanoma cells persist in a quiescent state under immune‐mediated and microenvironmental control before reactivation, highlighting the biologically unpredictable behaviour of melanoma and the importance of long‐term surveillance even after apparently definitive therapy [7].

Management of high‐risk melanoma, particularly in elderly patients, requires individualised decision‐making. Although adjuvant immunotherapy is increasingly recommended for stage IIC disease, a conservative surveillance approach was adopted following multidisciplinary discussion, as the patient declined systemic therapy due to concerns regarding toxicity in the context of advanced age [8]. At the time of pleural recurrence, the extensive nature of the disease and the patient's frailty guided the decision to pursue best supportive care rather than systemic therapy.

The prognosis for patients with stage IIC melanoma who decline adjuvant therapy is guarded, as high‐risk melanomas have significant potential for recurrence and metastasis. Studies suggest that the 5‐year melanoma‐specific survival for stage IIC disease is approximately 82%, but atypical sites of recurrence, such as pleural metastases, may occur and often indicate more aggressive disease behaviour [9]. Despite these risks, timely palliative interventions, including talc pleurodesis, can improve symptom control and quality of life, particularly in elderly or frail patients where systemic therapy may not be feasible.

This case highlights several key clinical lessons. Acral lentiginous melanoma can recur with atypical metastatic patterns, including predominant pleural involvement, and pigmented pleural effusions should prompt early consideration of melanoma metastasis in patients with relevant oncological histories. Medical thoracoscopy is invaluable for direct visualisation and targeted biopsy, facilitating definitive diagnosis when cytology is non‐diagnostic. Finally, management decisions in advanced melanoma must balance therapeutic benefit with patient frailty and treatment burden, underscoring the importance of individualised, patient‐centred care.

In conclusion, this case describes an uncommon manifestation of ALM with delayed pleural metastasis, highlighting the unpredictable metastatic behaviour of high‐risk melanoma. It underscores the potential for late recurrence despite prior definitive treatment and reinforces the importance of sustained clinical vigilance during long‐term follow‐up.

## Author Contributions

Ad Rian Chong, Khai Lip Ng, and Nai‐Chien Huan contributed to the design and implementation of the case report. Ad Rian Chong, Khai Lip Ng, and Nai‐Chien Huan drafted the manuscript. Ad Rian Chong, Khai Lip Ng, and Nur Husna Mohd Aminudin were involved in the clinical management of the patient, including the procedures and treatment described. Fazilah Hassan interpreted the histopathological findings and contributed to the histopathological analysis. Kasuma Mohamed Nordin supervised the project. All authors reviewed, discussed, and approved the final manuscript.

## Consent

The authors declare that written informed consent was obtained for the publication of this manuscript and accompanying images using the form provided by the Journal.

## Conflicts of Interest

The authors declare no conflicts of interest.

## Data Availability

Data sharing not applicable to this article as no datasets were generated or analysed during the current study.
